# Supplementary material for: Prenatal and postnatal droughts interact in shaping cognitive development
Source: Commun Med (Lond). 2026 Apr 20;6:233. doi: 10.1038/s43856-026-01578-7 (PMC13096533; doi:10.1038/s43856-026-01578-7)
Supplement: Supplementary file 2 — Supplemental Information [file 43856_2026_1578_MOESM2_ESM.pdf]

Supplementary Information (Online Appendix)

Prenatal and postnatal droughts interact in shaping cognitive development

Fabienne Pradella[1 2 3], Sabine Gabrysch[2 4 5], Reyn van Ewijk[1]

[1] Johannes Gutenberg University Mainz, Chair of Statistics and Econometrics, Mainz, Germany

[2] Heidelberg Medical Faculty and University Hospital, Heidelberg Institute of Global Health, Heidelberg, Germany

[3] Stanford University, Division of Primary Care and Population Health, Stanford, USA

[4] Charité – Universitätsmedizin Berlin, corporate member of Freie Universität Berlin and Humboldt-Universität zu Berlin, Institute of Public Health, Berlin, Germany

[5] Potsdam Institute for Climate Impact Research, Member of the Leibniz Association, Potsdam, Germany

Corresponding author

Fabienne Pradella, pradella@uni-mainz.de

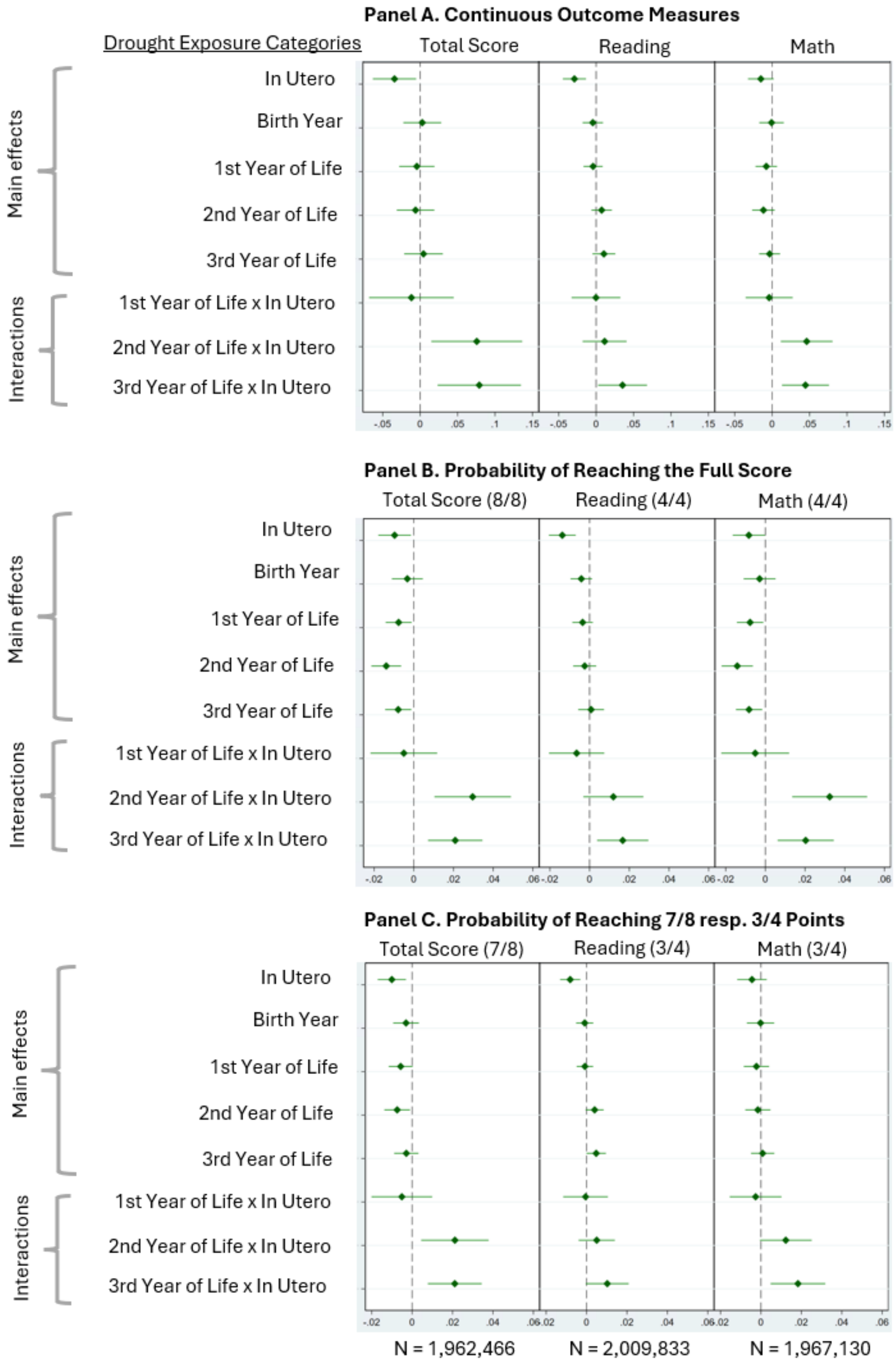

Fig. SI 1. Robustness check of main and interaction effects of prenatal and early-life drought exposure on adolescent cognitive function (ages 11–16, rural India): year of assessment fixed effects instead of time trend.

Forest plot displaying the effects of prenatal and early-life drought exposures on cognitive function. Within each panel, Total Score, Reading, and Math represent results from separate regression models, each corresponding to the outcome category indicated in the panel title. The coefficients of interest are the interaction terms between prenatal and early-life exposure (In Utero × 1st, 2nd, or 3rd Year of Life). All estimates are adjusted for child sex, age at assessment and year of assessment fixed effects.

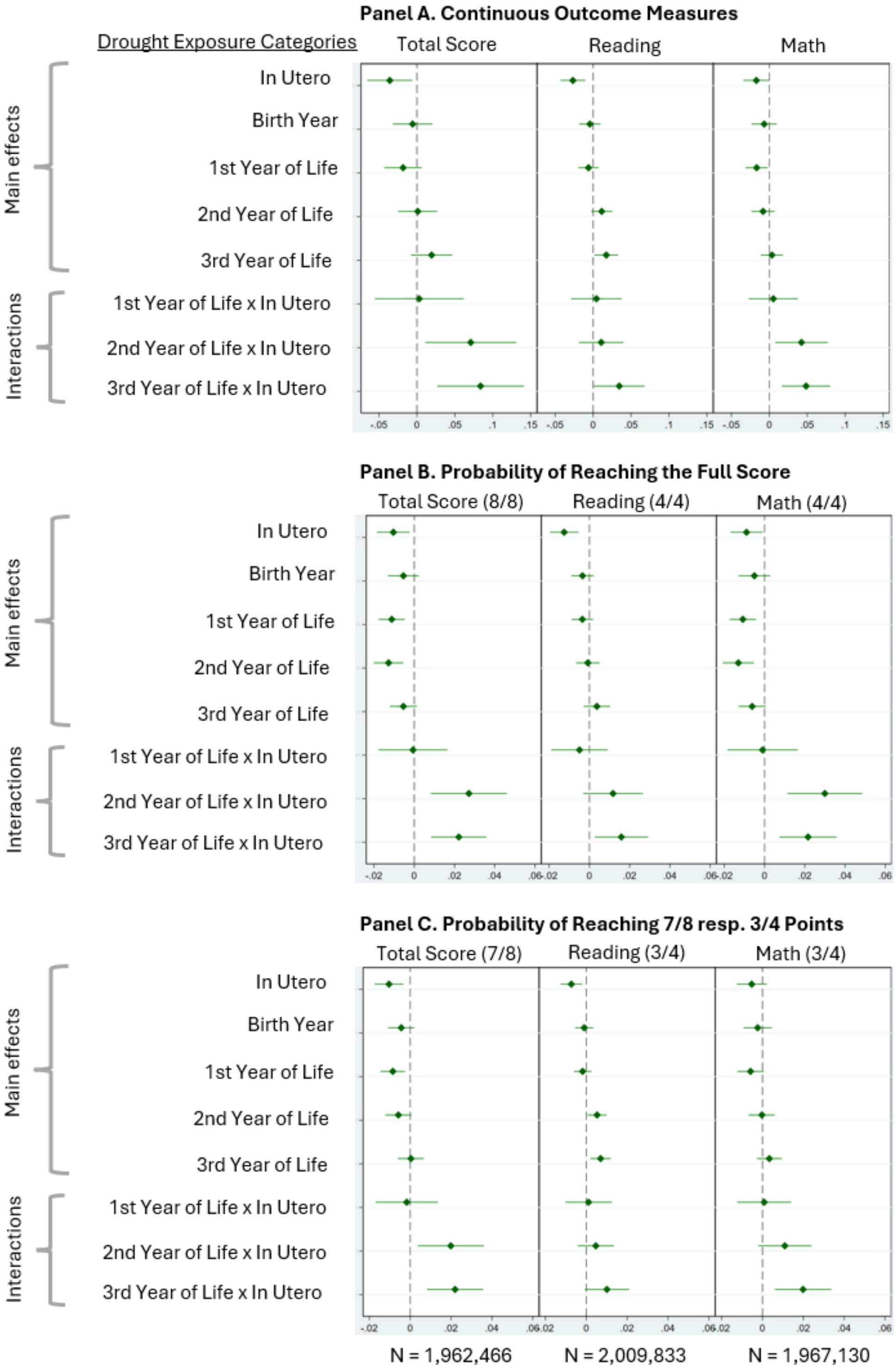

Fig SI 2 Robustness check of main and interaction effects of prenatal and early-life drought exposure on adolescent cognitive function (ages 11–16, rural India): additional adjustment for exposure to heavy rainfall (>95th percentile of district-specific long-term rainfall).

Within each panel, Total Score, Reading, and Math represent results from separate regression models, each corresponding to the outcome category indicated in the panel title. The coefficients of interest are the interaction terms between prenatal and early-life exposure (In Utero × 1st, 2nd, or 3rd Year of Life). All estimates are adjusted for child sex, age at assessment, a time trend (year of assessment and its square) as well as dummies for heavy rainfall at all early life stages (in utero, birth year, first year of life, second year of life, third year of life) measured as rainfall above the 95th percentile of the district-specific long-term rainfall.

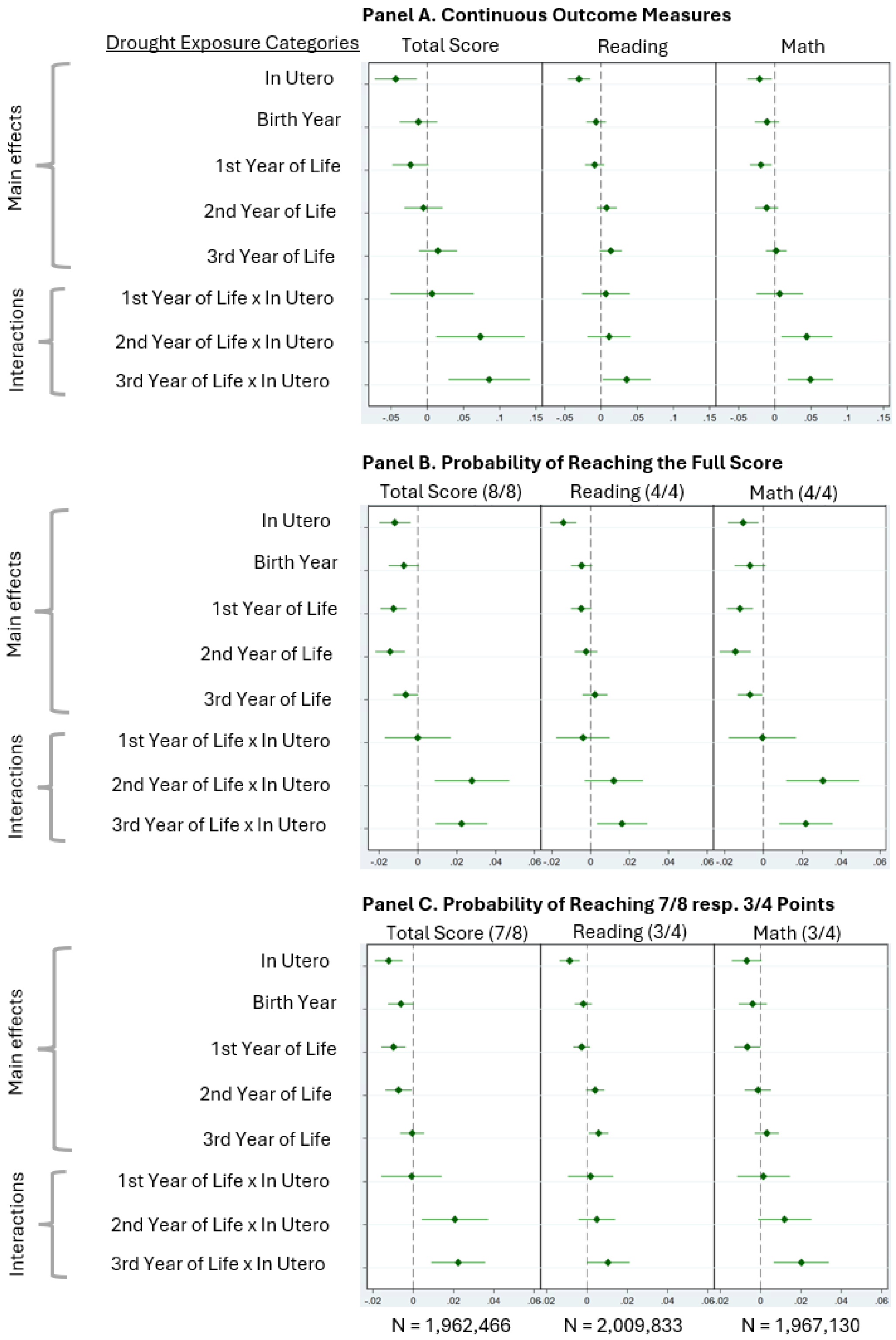

Fig. SI 3 Robustness check of main and interaction effects of prenatal and early-life drought exposure on cognitive function among 11–16-year-old adolescents in rural India: additional adjustment for firstborn status.

Within each panel, Total Score, Reading, and Math represent results from separate regression models, each corresponding to the outcome category indicated in the panel title. The coefficients of interest are the interaction terms between prenatal and early-life exposure (In Utero × 1st, 2nd, or 3rd Year of Life). All estimates are adjusted for child sex, age at assessment, a time trend (year of assessment and its square) as well as firstborn status.

Table SI 1 Negative control outcome analysis

Drought exposure category	Mother attended school (> 4 years)	Maternal highest grade completed (if went to school)	Maternal age at birth
In Utero	0.0003<br>(-0.0053 ; 0.0059)	-0.0324<br>(-0.0800 ; 0.0152)	0.0163<br>(-0.0428 ; 0.0753)
Birth Year	-0.0018<br>(-0.0083 ; 0.0047)	-0.0377<br>(-0.0864 ; 0.0109)	0.0038<br>(-0.0562 ; 0.0637)
First Year of Life	-0.0022<br>(-0.0087 ; 0.0042)	-0.0186<br>(-0.0614 ; 0.0242)	-0.0148<br>(-0.0695 ; 0.0400)
Second Year of Life	-0.0059<br>(-0.0129 ; 0.0011)	-0.0201<br>(-0.0634 ; 0.0232)	-0.0053<br>(-0.0583 ; 0.0476)
Third Year of Life	-0.0073<br>(-0.0146 ; 0.0001)	-0.0096<br>(-0.0483 ; 0.0291)	0.0118<br>(-0.0420 ; 0.0655)
Observations	4,402,169	2,149,778	4,248,719

This table displays the results of analyses in which maternal characteristics were regressed on the drought exposure categories. 95% confidence intervals are displayed in brackets. The regressions were additionally adjusted for a time trend (year of assessment and its square), child sex and district fixed effects. Asterisks are used to flag conventional levels of significance for ease of orientation (*** p<0.01, ** p<0.05, * p<0.1).

Table SI 2 Prenatal and early-life drought exposures: main effects and interaction effects on cognitive function among 11-16-year-old adolescents in rural India

	Continuous Outcome Measures			Probability of Reaching the Full Score			Probability of Reaching 7/8 resp. 3/4 Points		
**Drought Exposure Category**	**Total Score**	**Math Score**	**Read Score**	**Total Score (8/8)**	**Math (4/4)**	**Reading (4/4)**	**Total Score (7/8)**	**Math (3/4)**	**Reading (3/4)**
**In Utero**	-0.0433***	-0.0209**	-0.0302***	-0.0119***	-0.0104**	-0.0140***	-0.0122***	-0.00691*	-0.00858***
	(0.0149)	(0.00881)	(0.00794)	(0.00409)	(0.00405)	(0.00349)	(0.00354)	(0.00379)	(0.00258)
	[0.004]	[0.018]	[0.000]	[0.004]	[0.011]	[0.000]	[0.001]	[0.069]	[0.001]
**Birth Year**	-0.0120	-0.0107	-0.00693	-0.00723*	-0.00687*	-0.00466*	-0.00614*	-0.00395	-0.00184
	(0.0133)	(0.00860)	(0.00696)	(0.00399)	(0.00409)	(0.00277)	(0.00330)	(0.00358)	(0.00218)
	[0.367]	[0.213]	[0.320]	[0.070]	[0.094]	[0.093]	[0.063]	[0.270]	[0.398]
**1st Year of Life**	-0.0225*	-0.0189**	-0.00858	-0.0124***	-0.0120***	-0.00467*	-0.00963***	-0.00656**	-0.00253
	(0.0127)	(0.00771)	(0.00680)	(0.00339)	(0.00345)	(0.00270)	(0.00306)	(0.00333)	(0.00216)
	[0.076]	[0.015]	[0.208]	[0.000]	[0.001]	[0.084]	[0.002]	[0.0497]	[0.241]
**2nd Year of Life**	-0.00570	-0.0115	0.00734	-0.0144***	-0.0146***	-0.00255	-0.00747**	-0.00145	0.00395*
	(0.0133)	(0.00805)	(0.00712)	(0.00388)	(0.00408)	(0.00300)	(0.00331)	(0.00333)	(0.00233)
	[0.669]	[0.153]	[0.303]	[0.000]	[0.000]	[0.396]	[0.025]	[0.664]	[0.0913]
**3rd Year of Life**	0.0130	0.00112	0.0126	-0.00668**	-0.00728**	0.00168	-0.00103	0.00273	0.00546**
	(0.0134)	(0.00728)	(0.00781)	(0.00331)	(0.00333)	(0.00329)	(0.00308)	(0.00305)	(0.00248)
	[0.332]	[0.878]	[0.108]	[0.044]	[0.029]	[0.610]	[0.738]	[0.370]	[0.0282]
**1st Year of Life x In Utero**	0.00330	0.00517	0.00481	-0.000767	-0.00116	-0.00483	-0.00172	0.000817	0.00126
	(0.0294)	(0.0163)	(0.0168)	(0.00863)	(0.00879)	(0.00706)	(0.00761)	(0.00664)	(0.00573)
	[0.911]	[0.752]	[0.775]	[0.929]	[0.895]	[0.494]	[0.821]	[0.902]	[0.826]
**2nd Year of Life x In Utero**	0.0747**	0.0447**	0.0118	0.0280***	0.0309***	0.0122	0.0209**	0.0121*	0.00507
	(0.0308)	(0.0179)	(0.0151)	(0.00972)	(0.00955)	(0.00763)	(0.00835)	(0.00672)	(0.00459)
	[0.016]	[0.013]	[0.434]	[0.004]	[0.001]	[0.109]	[0.0126]	[0.0735]	[0.269]
**3rd Year of Life x In Utero**	0.0877***	0.0503***	0.0366**	0.0229***	0.0224***	0.0168**	0.0229***	0.0206***	0.0108**
	(0.0286)	(0.0159)	(0.0169)	(0.00674)	(0.00698)	(0.00665)	(0.00681)	(0.00694)	(0.00546)
	[0.002]	[0.002]	[0.030]	[0.001]	[0.001]	[0.012]	[0.001]	[0.003]	[0.0486]
Observations	1,962,466	1,967,130	2,009,833	1,962,466	1,967,130	2,009,833	1,962,466	1,967,130	2,009,833

This table presents the regression estimates displayed in Fig 1 (t-tests for statistical significance, two sided). Each column reports results from a separate model, the outcome is indicated in the header. The coefficients of interest are the interaction terms (*In Utero × 1st, 2nd, or 3rd Year of Life*). They are highlighted with a black box for ease of reference. All models adjust for child gender, age at assessment, and a time trend (year of assessment and its square). Coefficients are displayed with (robust standard errors) and [p-values] in parentheses. For ease of orientation, asterisks are used to flag conventional levels of significance (*** p<0.01, ** p<0.05, * p<0.1).
